# PES-Kaolin Mixed Matrix Membranes for Arsenic Removal from Water

**DOI:** 10.3390/membranes7040057

**Published:** 2017-09-30

**Authors:** Tiziana Marino, Francesca Russo, Lina Rezzouk, Abderrazak Bouzid, Alberto Figoli

**Affiliations:** 1 Institute on Membrane Technology, ITM-CNR, Via P. Bucci 17C, 87036 Cosenza, Italy; t.marino@itm.cnr.it (T.M.); ru.francy88@hotmail.it (F.R.); 2 Materials and Electronic Systems Laboratory (LMSE), University of Bordj Bou Arreridj, El-Anasser 34030, Bordj Bou Arreridj, Algeria; lina.r@live.fr (L.R.); a_bouzid34@hotmail.com (A.B.)

**Keywords:** arsenic, kaolin KT2, phase inversion, polyethersulfone, mixed matrix membranes, water treatment

## Abstract

The aim of this work was the fabrication and the characterization of mixed matrix membranes (MMMs) for arsenic (As) removal from water. Membrane separation was combined with an adsorption process by incorporating the kaolin (KT2) Algerian natural clay in polymeric membranes. The effects of casting solution composition was explored using different amounts of polyethersufone (PES) as a polymer, polyvinyl-pyrrolidone (PVP K17) and polyethylene glycol (PEG 200) as pore former agents, *N*-methyl pyrrolidone (NMP) as a solvent, and kaolin. Membranes were prepared by coupling Non-solvent Induced Phase Separation and Vapour Induced Phase Separation (NIPS and VIPS, respectively). The influence of the exposure time to controlled humid air and temperature was also investigated. The MMMs obtained were characterized in terms of morphology, pore size, porosity, thickness, contact angle and pure water permeability. Adsorption membrane-based tests were carried out in order to assess the applicability of the membranes produced for As removal from contaminated water. Among the investigated kaolin concentrations (ranging from 0 wt % to 5 wt %), a content of 1.25 wt % led to the MMM with the most promising performance.

## 1. Introduction

Arsenic (As) is a natural element, which behaves like a dangerous agent to human health and the environment. Several studies have shown a relationship between As exposure and teratogenicity, mutagenicity carcinogenic effects [1,2]. The harmful effects concern skin, bladder, lung liver, prostate cancer, as well as cardiovascular, pulmonary, immunological, neurological and endocrine diseases [3]. Drinking water represents the most As polluted source, especially in countries such as Argentina, Banghadesh, China, Chile, India and Mexico [4,5]. The World Health Organization [6] has set the maximum concentration limit to 10 μg/L or 10 ppb [6]. Arsenic exists in two primary forms: inorganic and organic. Inorganic compounds occur in two valence states (arsenite As(III) and arseniate As(V)), while organic compounds mainly are monomethyl arsenic acid and dimethyl arsenic acid [7]. Arsenic removal from water has been carried out in different ways. Classic options include coagulation, ion exchange, adsorption and membrane-based operations. Although these techniques are suitable for As removal, some of them still present disadvantages. In fact, coagulation requires a pre-oxidation step [8]. The efficiency of ion exchange strongly depends on the pH solution and concentration of anions [9,10]. Adsorption may represent one of the most promising removal process, offering the possibility to operate with high-removal efficiency, lower cost, simplicity, easy operation [11,12]. Molecules are removed from the aqueous solution by adsorption into solid surfaces. However, as reported in literature [8] this process is highly pH sensitive and requires periodical adsorbents regeneration. Moreover, this technique needs the recovery of adsorbents particles, contributing to the increase of cost maintenance [13]. Membrane technology should offer a promising solution for water treatment, especially when coupled with other removal techniques [5,14,15,16]. In this context, the mixed matrix membranes (MMMs) are particularly interesting; they pose a valid alternative, combining the positive properties of polymeric membrane and adsorbents. A MMM is composed of inorganic filler particles embedded in the polymeric matrix. Gohari et al. [17] and Zheng et al. [18] highlighted that the presence of adsorbents inside the membrane bulk should improve the separation selectivity, decrease fouling and increase membrane hydrophilicity. Mohan and Pittman [19] identified different adsorbents to remove As, such alumina, iron oxide, manganesia, titania and ferric phosphate. Metal oxides have large adsorption capacities due to their extremely high surface areas [20]. Fe(II) is present in different natural materials, such as magnetite, siderite and hematite, which have been investigated for removing As from water. The removal mechanism is mainly based on electrostatic attraction and adsorption on the surface of Fe(II)-bearing minerals [21,22]. Mohan and Pittman [19] studied the possibility of using activated carbon, showing that As(V) can be removed more efficiently than As(III). Among the naturally occurring materials, kaolinite, montmorillionite and illite [23,24] represent particularly attracting clay minerals with good adsorption capabilities. Kaolinite is a group of alumina silicates clay minerals which comprise the principal ingredients of kaolin and it is characterized by fine particle size, brightness and whiteness, chemical inertness, structure [24].

“KT2” (Table 1), is an Algerian kaolin coming from the original EL Milia deposit “TAMAZERT” in the region of Jijel (Algeria). This kaolin is abundant in soils and sediments [25], it is odorless white to yellowish or grayish powder and it has density 2.65 g/cm^3^ [26]. It has been selected for water treatment studies for its high capacity adsorbents on the surface, very low cost material and alumino silicate-based compositions (Al_2_O_3_(SiO_2_)_2_(H_2_O)_2_) [24,25,27,28,29,30,31].

Zen et al. [28] tested two Algerian kaolin clays, DjebelDebagh “DD3” and Tamazert “KT2” for the adsorption of Derma Blue R67 acid dye, commonly used in the tanning industry. The results obtained evidenced the efficiency and the feasibility of dye adsorption at ambient temperature, thus demonstrating that natural clays work as promising adsorbent candidates for waste water treatments.

Sarbatly [30] described the preparation of kaolin–polyethersufone (PES) membranes via NIPS and sintering techniques, investigating the effect of the clay/polymer ratio and sintering temperature on pore size and porosity. The registered data showed that the pore size changed from 20 to 8 μm and the porosity decreased from 26% to 11% when additive/polymer and sintering temperature increased from 1 to 3.5 and from 1100 to 1500 °C, respectively.

Han et al. [31] worked on the production of Al_2_O_3_, Al_2_O_3_–SiO_2_ and Al_2_O_3_–kaolin hollow fibers using a wet-spinning method using PES as polymeric material. Hollow fiber membranes were prepared by combining preheating and sintering processes. The addition of SiO_2_ was beneficial for Al_2_O_3_ hollow fiber membranes, even if the amount of SiO_2_ should be rigorously controlled. When kaolin was used instead of SiO_2_, the introduction of both the reactions of Al_2_O_3_–SiO_2_ were achieved. In fact, kaolin, acting as an inorganic binder, contributed to the sintering of the membranes, thus the structure was maintained and the particles fixed up by the reaction of alumina and silica in kaolin.

In this work, kaolin was chosen as a natural adsorbent for the preparation of a MMM flat sheet membrane using PES as polymeric material.

The adsorption capability of the natural clay was tested for As removal from water. Considering the outstanding property of kaolin as an inorganic binder in the membrane preparation process, its natural abundance, fine texture, grain size of 0.2–1 μm, high chemical and thermal stability and low cost, kaolin should be adopted in membrane preparation as substitute of Al_2_O_3_, TiO_2_ and ZrO_2_ [28,29,30,31].

PES is among the most used amorphous thermoplastics which finds applications in many industrial sectors, such as electronic, medical, aerospace, food service [32]. It presents excellent thermal and chemical stability, dimensional stability and resistance in a wide range of pH [33].

The aim of this work was the fabrication and the characterization of organic-inorganic PES–kaolin membranes for arsenic removal from water. Membrane separation was coupled with the adsorption process by including Algerian natural clay (kaolin KT2) into the polymeric solution.

MMMs were prepared by coupling NIPS (Non-solvent Induced Phase Separation) and VIPS (Vapour Induced Phase Separation) procedures in order to study the influence of several parameters which affect the final membrane structure and properties, such as kaolin and pore former agent content, as well as the exposure time to relative humidity.

## 2. Materials and Methods

### 2.1. Materials

Polyethersulfone (PES) polymer (Veradel^®^3000 P; Mw = 60 kg/mol) was kindly supplied by Solvay Specialty Polymers (Bollate, Italy) and *N*-methyl pyrrolidone (NMP) synthetic grade was purchased by Sigma Aldrich (Milan, Italy).

Polyvinylpyrrolidone (PVP, Luviskol K17; Mw = 9 kg/mol) was purchased by BASF (Ludwigshafen, Germany) and as well as for the polymer, was desiccated under vacuum at 50 °C for 24 h before its use. Polyethylene glycol (PEG; Mw = 0.2 kg/mol) was purchased by Sigma Aldrich. The precipitation medium was *i*-distilled water (at 15 °C). Kaolin (KT2), was obtained from Guelma region (Algeria).

### 2.2. Membrane Preparation

Membranes were prepared via phase inversion, based on the separation of an initially stable solution, in a polymer-rich phase and a polymer poor-phase [34].

Thermodynamic instability during phase inversion can be induced by:

- Adding a non-solvent (non-solvent induced phase separation, NIPS).
- Exposing the polymeric solution to a non-solvent vapour (vapour induced phase separation, VIPS) up to the complete polymer precipitation or prior to immersion in a non-solvent coagulation bath.
- Evaporating the solvent (evaporation induced phase separation, EIPS).
- Cooling down a polymer solution obtained at elevated temperature promoting polymer precipitation and the formation of the final membrane (thermally induced phase separation, TIPS).

In this work, membranes were produced via NIPS or by coupling NIPS and VIPS.

In particular, MMMs with different morphology and properties were prepared by changing in the dope solution the kaolin and the PEG content, and by varying the exposure time of the nascent film to humid air (Table 2).

The optimized procedure for preparing casting solution was the following:

1. The liquid phase (NMP and PEG) was magnetically stirred.
2. Kaolin was added and the suspension sonicated in an ultrasonic bath (for 90 min at 25 °C) in order to assure a homogeneous nanoparticles dispersion.
3. The solid components (i.e., PES and PVP) were added and kept under stirring until a homogeneous casting suspension was observed.

The dope solution were cast on a glass plate using a manual casting knife (Elcometer 3700/1 Doctor Blade, Aalen, Germany; adjustable gap size: 30–4000 μm) with a 300 μm gap, at 25 °C and 55% of relative humidity (RH %) in a climatic chamber (DeltaE srl, Rende, Italy).

Membranes were washed three consecutive times with hot water (60 °C), dried at room temperature for 4 h and then dried in an oven at 40 °C for 24 h before characterize them.

### 2.3. Experimental Set up

**Figure 1:**
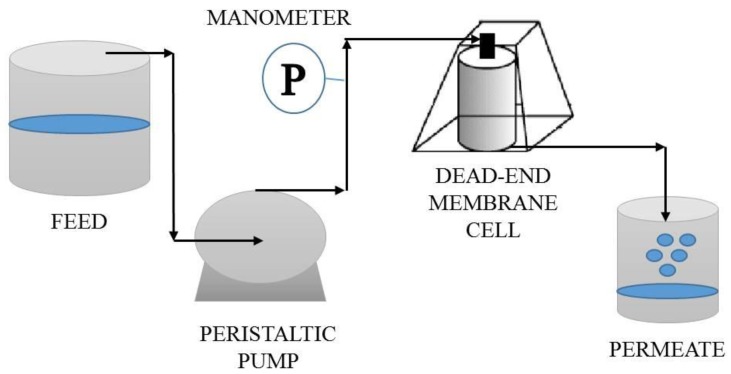
Experimental set up used for Arsenic (As) removal from aqueous solutions.

The experimental set-up is composed of Amicon Model 8200 (EMD Millipore, Billerica, MA, USA), equipped with a filtration unit (180 mL). 1 ppm of Sodium arsenate dibasic heptahydrate was placed into 200 mL of water. As aqueous solution was forced to pass through the membrane by means of a peristaltic pump (Masterflex^®^7518-10, Cole-Parmer, Vernon Hills, IL, USA) (Figure 1).

Both the feed and the permeate composition were evaluated by Inductively Coupled Plasma (ICP) analysis (performed by GEOLAB S.r.l. laboratory, Rende, Italy) after specific contact time. Before ICP analysis, all the samples were stabilized with ultrapure nitric acid (1.0% HNO_3_).

The percentage of Arsenic removal %*R* was determined as follows:

%*R* = ((*C*_0_ − *C_t_*) × 100)/*C*_0_ (1)

where *C*_0_ is the initial As concentration and *C_t_* is the As content at time *t*.

### 2.4. Pure Water Permeability (PWP)

The membrane performance was tested in a cross flow system in recycle mode. In this set up permeate is pumped back to the feed water solution. The permeate flux was determined by monitoring the permeate weight during time. PWP was measured at 25 °C by means of a cross-flow cell (DeltaE srl, Italy). Pure water at 25 °C was driven across the membrane with 8 cm^2^ area, by using Tuthill Pump Co. (Alsip, IL, USA) gear pump. *PWP* was calculated using the following equation:

(2) $$PWP=\frac{Q}{A\;t\;\Delta P}$$

where *Q* is the permeate volume (L), *A* is the membrane area (m^2^), *t* is the time (h), and Δ*P* is the pressure difference across the membrane sides.

Three different transmembrane pressures (i.e., 1.0/0.8/0.6 bar separated by a stabilization period of one to another of 20 min), were used to calculate PWP.

### 2.5. Thickness

The thickness was determined by the multiple point method using a digital micrometer (Carl Mahr, Göttingen, Germany; precision of ±0.001 mm). Thickness was verified for seven regions of membrane, the average value and the standard deviation were taken into account.

### 2.6. Porosity

Membrane porosity was determined by cutting three different samples of the same membrane, drying them in a vacuum oven at 50 °C for 12 h and measuring their weight by means of an analytical balance. Then, the samples were soaked in Kerosene for 24 h before verifying the wet membrane weight. The average and the standard deviation values were then calculated. Membrane porosity (ε) is defined as the volume of the pores divided by the total volume of the membrane. The porosity was calculated by the following equation [35]:

(3) $$\varepsilon =\frac{\frac{{\mathrm{wt}}_{w}-{\mathrm{wt}}_{d}}{\rho_{k}}}{\frac{{\mathrm{wt}}_{w}-{\mathrm{wt}}_{d}}{\rho_{k}}+\frac{{\mathrm{wt}}_{d}}{\rho_{p}}}\times 100$$

where ε is the porosity (%), wt_w_ and wt_d_ are the wet and dry weight of the membrane, respectively; ρ_k_ and ρ_p_ are the density of kerosen and PES, respectively.

### 2.7. Pore Size and Pore Size Distribution

The analysis was performed three times for each membrane, and by extrapolating the average and the standard deviation. A capillary flow porometer (CFP-1500 AEXL, Porous Materials Inc., Ithaca, NY, USA) was used. Dried membranes were kept in a wetting liquid (Fluorinert^®^FC-40, Sigma-Aldrich) having low surface tension (16 dynes/cm) for 24 h prior analyzing the pore size.

The microflow porometer measures pressure close to the membrane. Differential pressures and gas flow rates through dry and wet samples were recorded. The pore structure characteristics include bubble point, mean flow pore size, pore fraction distribution and air permeability.

### 2.8. Mechanical Properties of Membranes

The mechanical properties which include the Young’s modulus and elongation at break of the prepared membranes were measured using a Zwick Roell Z2.5 test unit (Zwick Roell Group, Ulm, Germany) with pneumatic clamps and 50 N maximum load cell. The stress (σ) calculated as the ratio between the applied force (*F*) per unit area (*A*).

While the strain is defined as:

ε = ln(*L*)/*L*_0_ (4)

where *L* is the sample length at break and *L*_0_ is the initial sample length, allow to calculate Young’s modulus (*E*) corresponding to: *E* = σ/ε from the initial part of the slope from the stress/strain curve [36]. Membranes were cut into strips of 1 cm width and 5 cm length. Tests were carried out by starting with an initial 50 mm gauge length and by stretching the membranes unidirectional at a constant rate of 5 mm/min. 5 specimens were used for each membrane and the average and the standard deviation were extrapolated.

Three samples (1 cm × 5 cm) of each type of membrane were tested at ambient temperature with a cross head speed of 0.5 mm/min. The tension strength and elongation at break average values as well as their standard deviations were determined.

### 2.9. Scanning Electron Microscopy (SEM) Analysis

Cross section and surface of the membranes produced were observed with a Zeiss-EVO MA10 instrument (Zeiss, Milano, Italy). The membranes were first freeze fractured using liquid nitrogen for cross section preparation. Then for both surface and cross-section analyses, the samples were sputtered for making a thin gold layer (by using a Quorum Q150 RS sputter (Quorum Technologies, Laughton, East Sussex, UK) in order to enhance membrane conductivity and prevent electrical charging.

### 2.10. Contact Angle

The static contact angle of water can be defined as the angle comprised between the membrane surface and the tangent line at the water droplet contact point on the membrane surface. An optical contact angle meter (CAM100, KSV Instruments, Helsinki, Finland) was used to measure the contact angle of the membranes prepared. A water droplet of 5 μL was positioned on the membrane top surface. For each membrane, five measurements were carried out, at the end of which the average and the standard deviation were calculated.

## 3. Results and Discussions

### 3.1. Membrane Morphology

**Figure 2:**
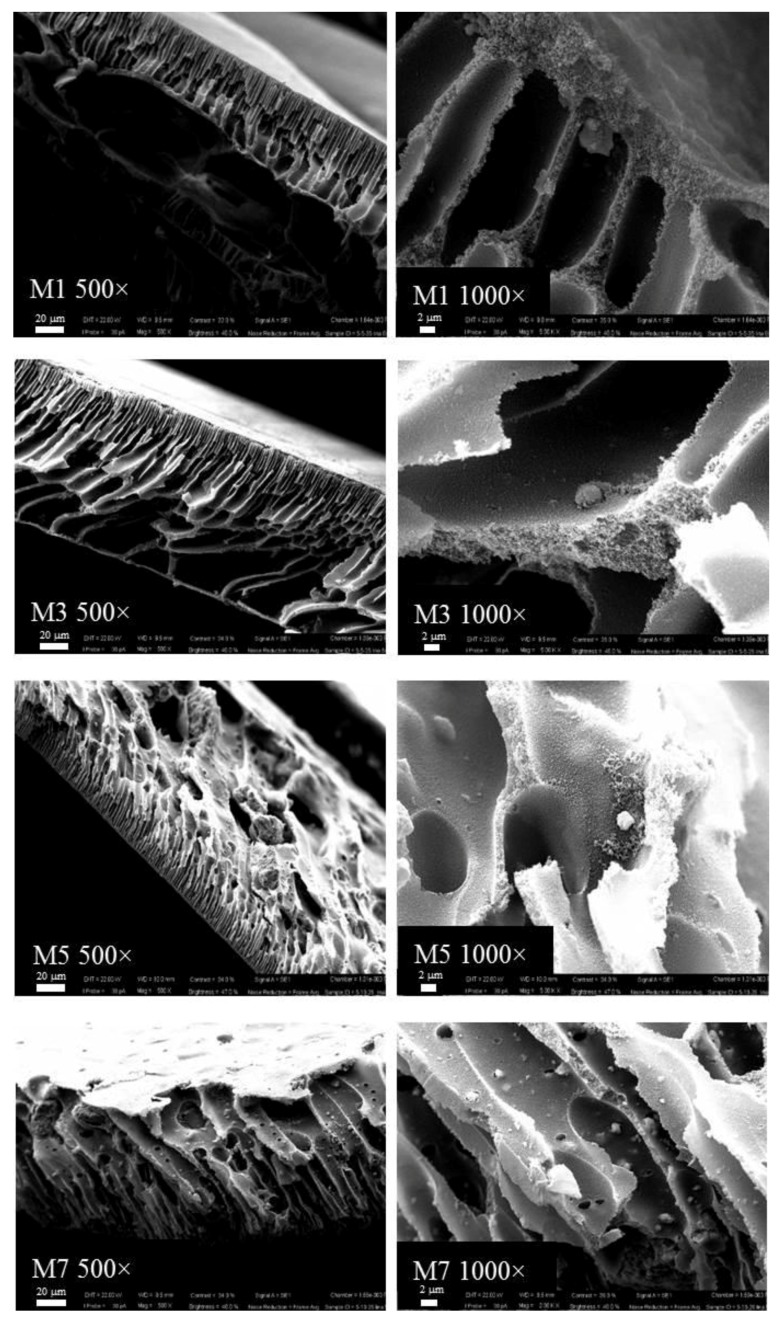
Scanning electron microscopy (SEM) images of the PES and PES–kaolin membranes prepared via the Non-solvent Induced Phase Separation (NIPS) procedure.

**Figure 3:**
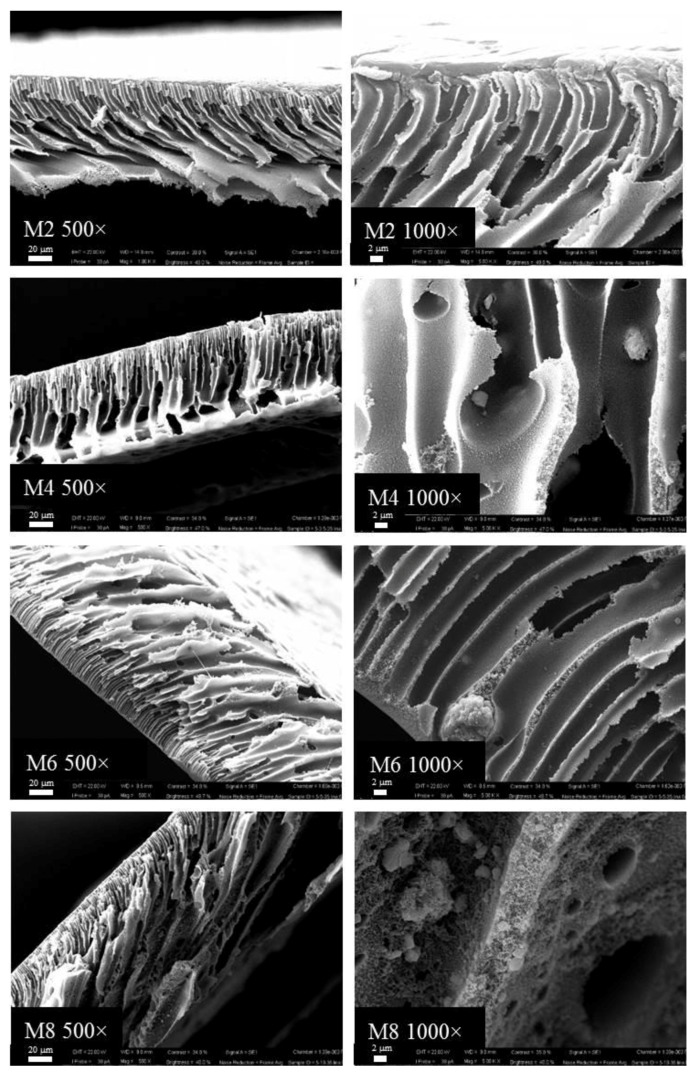
SEM images of the PES and PES–kaolin membranes prepared via NIPS–Vapour Induced Phase Separation (VIPS) procedure.

The influence of the concentration of kaolin, as well as the adopted membrane preparation procedure, was investigated using scanning electron microscopy (SEM) apparatus. The SEM images of the cross-sections of pristine PES and MMMs membranes prepared with different concentrations of kaolin via NIPS are represented in Figure 2. PES membrane (M1) exhibited a typical asymmetric structure, composed of a thin skin layer and a porous bulk with a finger-like structure. Similarly to what was observed for the pristine PES membrane, by increasing the kaolin content in the dope solution, the membrane exhibited porous finger-like morphology (Figure 3). The bounding of PES chains and kaolin nanoparticles could be observed with further SEM magnification (1000×), which showed that the kaolin particles were entrapped in the membrane structure by means of interactions with polymer chains.

By coupling the NIPS and VIPS procedures, M2, M4, M6 and M8 membranes were obtained by changing the kaolin concentration (Figure 3).

During VIPS process, the cast film is first exposed to humidity prior to immersion in the coagulation bath. The non-solvent in liquid and vapour form leads to the phase separation. Typically, by adjusting some parameters, such as temperature, relative humidity and exposure time, membranes with large pores on the top surface can be obtained [37].

Although the presence of non-solvent in the vapour phase may reduce the exchange rate between solvent and non-solvent, leading to sponge-like symmetric structures, the presence of hydrophilic kaolin nanoparticles may contribute to the faster water–NMP demixing, promoting the formation of fingers.

Kaolin nanoparticle dispersion was visibly homogeneous without evident defects across the membrane morphology. However, as reported in the literature [31], a small number of aggregations formed with nanoparticles with particularly high surface would contribute to reduce the surface energy, thus enhancing the system stability.

All the membranes prepared displayed a very compact polymeric layer on the top and the bottom surface, with few bare kaolin particles. This might be related to the intrinsic binder properties of kaolin [38]. In fact, it is used in ceramic preparation thanks to its tremendous molding performance. At lower kaolin content, inorganic binding of the membranes could be established by inorganic nanoparticles, promoting membrane formation and particle dispersion. However, at higher concentrations, kaolin would negatively affect the particle dispersion, giving rise to the formation of some defects in the membrane structure [31].

### 3.2. Membrane Pore Size

In order to achieve separation, membrane systems rely on membranes which are able to remove undesirable compounds or allow the passage only of selected kinds of solutes. It is evident that pore size represents an important parameter to achieve this separation. The pore size of the produced PES–kaolin membranes is reported in Table 3.

Membranes prepared without kaolin (M1 and M2) present noticeable differences in the pore size depending on the preparation method adopted. In particular, the mean flow pore diameter was lower (0.05 ± 0.01 μm) when the membrane was produced via NIPS and increased up to 0.14 ± 0.01 μm by exposing the nascent film to controlled humid air and temperature for 5 min before immersion in the water bath (NIPS–VIPS procedure). In accordance with what has been reported in the literature [39], the non-solvent in vapour form promoted a reduction in the polymer precipitation rate, due to a delayed solvent/non-solvent exchange, thus allowing the growth of membrane pores.

The addition of kaolin in the casting solution further promotes the increase in membrane pore size, due to the increase in dope solution viscosity, which changed in the following order: 1120 cP (0 wt % kaolin) > 1640 cP (1.25 wt % kaolin) > 2130 cP (2.50 wt % kaolin) > 3550 cP (5 wt % kaolin). The increased viscosity hindered the exchange process between water in the coagulation bath and NMP in polymer dope, enhancing, at the same time, water inflow rate in the membrane matrix. Similar results were reported by Ma et al. [40].

Pore formation is further supported by the presence of the non-solvent in vapour form, which caused the increase in the pore size when NIPS was coupled with VIPS. In fact, during VIPS, the presence of water in vapour form was found to strongly affect membrane formation, especially in cases where water represents a strong non-solvent for the polymer and the affinity with the solvent is high (such in this case for the water–NMP system) [41]. By increasing the exposure time to humid air, membranes with higher pore size could be produced [39].

In this case the pore size changed from 0.14 ± 0.01 μm (M2) to 0.17 ± 0.01 μm (M4) and 0.23 ± 0.01 μm (M6) up to 0.26 ± 0.01 μm (M8) by increasing the kaolin content in the dope solution.

### 3.3. Thickness, Porosity, Contact Angle and Mechanical Properties

Thickness, porosity, contact angle and mechanical features are key parameters, which strongly influence the efficacy of separation operations and are correlated to the membrane morphology and PWP. Membrane thickness is a significant factor affecting the separation rate across the membrane. As presented in Table 4, the thickness of the membranes prepared with and without kaolin was almost unchanged, even when the nascent films were exposed to controlled humidity and temperature before polymer precipitation in the coagulation bath.

The contact angle is an important parameter, which proves the hydrophilic or hydrophobic membrane characteristic [42,43]. The contact angles of the MMMs obtained are reported in Table 4. By preparing membrane via NIPS, the addition of kaolin nanoparticles into the polymeric solution, resulted in an increase in contact angle from about 60° for the control membrane (M1) to 70° (M7) for the highest investigated kaolin content (5.00 wt %). Similarly, also by producing membranes via NIPS–VIPS, membranes exhibited increasing contact angle values by increasing kaolin concentration in the dope solution. In this case, the contact angle passed from 65° (M2) to 70° (M8). These results may be related to increases in membrane roughness, and, as reported by Mierzwa et al. [33], the increase in contact angle may also depend on nanosize effects. However, a more in-depth investigation on factors affecting membrane hydrophilicity would be necessary to completely understand the effect of kaolin on the final membrane properties. Mierzwa et al. [33] also reported the preparation of PES–clay ultrafiltration membranes via NIPS, varying the clay content in the range between 1 wt % and 5 wt %. In agreement with what was observed in this work, the contact angles of the membranes prepared increased with the increase in clay content. However, in other studies clay-doped membranes were reported to have lower or similar contact angle to the undoped polymeric membranes. Anadao et al. [44] described the preparation and the characterization of nanocomposite polysulfone (PSf) and sodium montmorillonite (MMT) membranes, for which the water contact angle slightly decreased with the increase in MMT content. Also Ghaemi et al. [45] reported the production of significantly more hydrophilic PES–organically modified MMT membranes when the clay mineral content increased, with corresponding increases in water flux. In contrast, Monticelli et al. [46] registered minor changes in contact angle for the membranes fabricated with pristine PSf and PSf–cloisite 93A mixtures.

The presence of kaolin had a positive effect on membrane porosity (Table 4). In fact, the porosity gradually increased with the increasing clay content in the polymeric solution, passing from 85.40 ± 0.18 for M1 to 90.57 ± 2.30 for M8. Similar observations were reported by Ma et al. [40], who attributed the porosity increase to the lower solvent-non solvent exchange rate which in turn was caused by the incorporation of clay nanoparticles in the polymeric matrix. The increased water inflow rate in the nascent film was responsible for the formation of more porous membranes. The membranes prepared via NIPS–VIPS procedure presented higher porosity than those obtained by NIPS. Also in this case the reasons may lie with the decreased demixing rate between NMP and water due to the delayed water vapour adsorption into the nascent membrane, which promote the formation of membranes with more pores and larger pore size.

Elastic modulus and elongation at break are two important factors to define the mechanical resistance of membranes. The effects of kaolin concentration on mechanical strengths of PES–kaolin membranes is shown in Table 4. By increasing the clay content, Young’s modulus decreased from 98.85 ± 1.69 (M1) to 70.94 ± 2.55 (M8) MPa, while the elongation at break passed from 2.48% ± 0.70% (M1) to 1.15% ± 0.36% (M8). The increased pore size and porosity of MMMs may induce worsening of the mechanical properties. Moreover, by increasing the kaolin dosage in the dope solution, the inhomogeneity of filler distribution inside the membrane matrix may lead to particle agglomeration, which further deteriorates membrane mechanical resistance. A similar effect was also reported by Ma et al. [40].

### 3.4. Pure Water Permeability (PWP)

**Figure 4:**
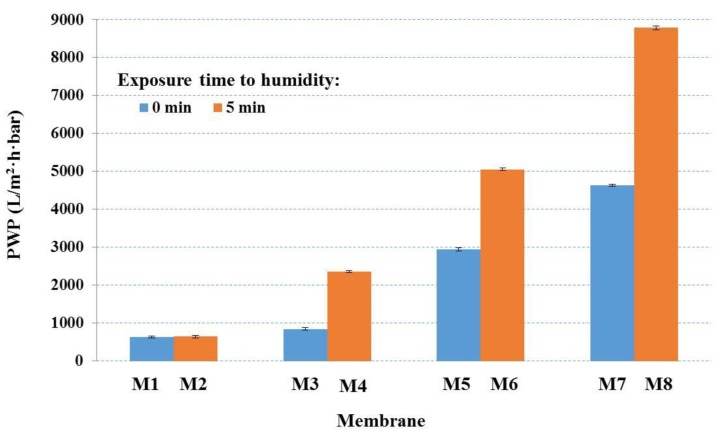
Pure water permeability (PWP) of the prepared PES and PES–kaolin membranes.

Pure water permeability (PWP), or pure water flux, represents a critical parameter for porous membranes, and it is directly correlated with membrane pore size and porosity [31,40,47]. Figure 4 shows the effects of kaolin content on PWP (average and standard deviation) for the membranes produced. PES membranes prepared via NIPS and NIPS–VIPS methods (M1 and M2, respectively) displayed similar PWP. In contrast, the preparation methods had a critical influence on PWP for the PES–kaolin membranes. In fact, MMMs exposed to controlled humid air and temperature before the immersion in the coagulation bath exhibited considerably higher PWP than those obtained via NIPS, and showed higher values than the pristine PES membranes. PWP changed from 843 ± 29 to 8781 ± 32 L/m^2^·h·bar for M3 and M8, respectively. The results obtained indicated that PWP reflected pore size and porosity data, but may be ascribed also to the presence of kaolin in the membrane matrix. Ma et al. [40] reported that the addition of clay in the polymeric solution led to an increase in the large pore ratio in the skin layer, which explained the registered increased pure water flux with the increase of clay dosage.

### 3.5. Membrane Performance Evaluation: As Removal from Water

**Figure 5:**
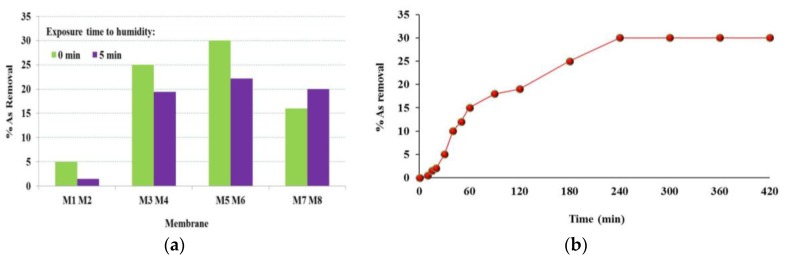
(**a**) As adsorption after 240 min test by using PES and PES–kaolin membranes prepared via NIPS and NIPS–VIPS; (**b**) Kinetic of As adsorption on the M5 PES–kaolin membrane.

In order to evaluate the applicability of PES–kaolin membranes on water treatment, As adsorption tests were carried out using 4 h as contact time. As reported by Zen et al. [28], As removal capacity is a parameter ruled by physical adsorption over the membrane, and the adsorption rate for the contact time is influenced by several parameters, such as the clay particle size entrapped into the membrane, the ionic strength and the size of As compounds. Figure 5a illustrates As adsorption capacity after 4 h for the unmodified PES membranes as well as for PES–kaolin membranes. It can be noted that As removal by adsorption through the PES membranes was very low (5% for M1 and 1.5% for M2). When kaolin was used, As removal significantly increased, reaching a value of up to 30% when 2.5 wt % kaolin was incorporated into the polymeric solution and the membrane was prepared via NIPS method (M5).

By using MMMs membranes, As removal firstly increased by increasing the clay dosage from 1.25 wt % to 2.5 wt %, then decreased when kaolin concentration was further increased up to 5 wt %. These results may be related to the active adsorbent sites of clay particles distributed in the membrane bulk. In fact, an initial rapid increase in the adsorption upon a higher kaolin dosage may be related to a higher number of As-adsorption available sites. In presence of 2.5 wt % kaolin, the removal of As reached the maximum value of 30%. However, by increasing the adsorbent load, there was a decrease in the As removal, which means that, among the kaolin dosages investigated, 2.5 wt % was the optimal amount of clay necessary to remove As from water. When a 5 wt % clay was used, the active sites were probably occluded due to the kaolin particles’ aggregation. A similar behavior was reported for Derma Blue R67 acid dye [48], cationic starch derivatives [49], methyl orange, Ponceau 6R, and Congo Red adsorbed on pullulan microspheres [50]. All the membranes prepared via NIPS presented a better performance in comparison to that of the analogue membranes obtained via NIPS–VIPS. This may be related to the combination of some membrane characteristics, such as morphology, pore size and porosity, but also PES–kaolin–As interactions that favored the arsenic adsorption in the membrane matrix. However, further investigations are needed to better understand how the operational conditions can affect the adsorption process.

On the basis of the adsorption results discussed above, the adsorption dynamic kinetics of As on M5 was analyzed (Figure 5b).

The adsorption capacity increased rapidly in the first hour (15% As removal after 60 min) and reached a value of 30% after 250 min. After this time, an adsorption equilibrium was observed, and the variation of As removal was maintained unchanged during the extended contact time (420 min). These observations may arise from the accessibility of kaolin bonding sites, which resulted completely hindered by the adsorbed As.

## 4. Conclusions

Flat sheet PES–kaolin membranes with different clay dosages were prepared by NIPS and NIPS–VIPS procedure. The membranes prepared exhibited asymmetric structure, in which the incorporation of well-dispersed kaolin nanoparticles can be clearly observed. The combination of the operational conditions adopted to produce the membranes allowed us to tailor their properties. The presence of kaolin promoted pore formation and the increase in its content led to an increase in the membrane pore size and PWP. In contrast, membrane hydrophilicity and mechanical resistance worsened when the clay was included in the polymeric solution. Preliminary tests on As removal from water were carried out, showing that inorganic-organic membranes had a better performance than the analogue PES membranes. Even though further investigations are needed to improve the membrane-based As adsorption, this work demonstrated that kaolin can be efficiently used as a low cost natural material for the preparation of MMMs to be used for water treatment.

## Figures and Tables

**Table 1:** Chemical composition of KT2 kaolin [28].

Components (wt %)								
SiO_2_	Al_2_O_3_	Fe_2_O _3_	MgO	CaO	Na_2_O	K_2_O	TiO_2_	LOI
49.30	33.50	1.59	0.40	0.08	0.09	2.75	0.24	10.50

**Table 2:** Compositions of the casting solutions and exposure time to controlled temperature (25 °C) and RH %.

Membrane Code	PES (wt %)	KT2 Kaolin (wt %)	PVP K17 (wt %)	PEG 200 (wt %)	NMP (wt %)	Exposure Time to RH % (min)
M1	12	0	5	35	48	0
M2						5
M3	12	1.25	5	35	46.75	0
M4						5
M5	12	2.5	5	35	45.5	0
M6						5
M7	12	5	5	35	43	0
M8						5

**Table 3:** Smallest, mean flow and largest pore diameter of the prepared membranes.

Membrane Code	Pore Size					
	Smallest Pore Size (μm)		Mean Flow Pore Diameter (μm)		Largest Pore Size (μm)	
	Average	Std. Dev.	Average	Std. Dev.	Average	Std. Dev.
M1	0.03	0.01	0.05	0.01	0.06	0.02
M2	0.13	0.02	0.14	0.01	0.15	0.02
M3	0.05	0.01	0.10	0.01	0.18	0.01
M4	0.06	0.02	0.17	0.01	0.23	0.03
M5	0.05	0.02	0.12	0.02	0.23	0.01
M6	0.08	0.02	0.23	0.01	0.31	0.01
M7	0.05	0.03	0.21	0.02	0.33	0.02
M8	0.02	0.01	0.26	0.01	0.32	0.02

**Table 4:** Thickness, porosity, contact angle and mechanical properties of the prepared PES and PES–kaolin membranes.

Membrane Code	Thickness (mm)	Porosity (%)	Contact Angle (°)	Mechanical Properties	
			Top Surface	Young’s Modulus (n/mm^2^)	Elongation at Break (%)
M1	0.099 ± 0.000	85.40 ± 0.18	52.30 ± 0.14	98.85 ± 1.69	2.48 ± 0.70
M2	0.094 ± 0.001	86.45 ± 0.99	66.11 ± 1.17	96.77 ± 1.04	2.62 ± 0.49
M3	0.103 ± 0.013	89.36 ± 0.45	66.48 ± 0.24	77.35 ± 1.72	2.81 ± 0.40
M4	0.099 ± 0.002	89.74 ± 0.69	62.04 ± 0.89	76.52 ± 1.88	2.29 ± 0.54
M5	0.109 ± 0.004	89.67 ± 0.00	71.97 ± 2.38	74.85 ± 2.54	2.81 ± 0.95
M6	0.110 ± 0.001	89.97 ± 0.43	75.32 ± 1.09	72.41 ± 2.09	2.94 ± 0.55
M7	0.111 ± 0.008	90.54 ± 0.78	73.80 ± 3.66	72.15 ± 0.75	1.17 ± 0.76
M8	0.100 ± 0.013	90.57 ± 2.30	67.26 ± 2.45	70.94 ± 2.55	1.15 ± 0.36
